# A Brother's Grimm true story: the valiant little triple-quadrupole - 7 allergens in one blow

**DOI:** 10.1186/2045-7022-1-S1-S82

**Published:** 2011-08-12

**Authors:** Bert Popping

**Affiliations:** 1 Germany

## 

The development of a multi-method for the detection of seven allergens based on liquid chromatography and triple-quadrupole tandem mass spectrometry in multiple reaction mode is presented. It is based on extraction of the allergenic proteins from a food matrix, followed by enzymatic digestion with trypsin. The chosen marker peptides were implemented into one method that is capable of the simultaneous detection of milk, egg, soy, hazelnut, peanut, walnut and almond. This method has been used to detect all seven allergenic commodities from incurred reference bread material, which was baked according to a standard recipe from the baking industry. The presentation demonstrates the high potential of LC-MS/MS for allergens screening for the food industry.

